# Flip-flop method: A new T1-weighted flow-MRI for plants studies

**DOI:** 10.1371/journal.pone.0194845

**Published:** 2018-03-29

**Authors:** Simon Buy, Simon Le Floch, Ning Tang, Rahima Sidiboulenouar, Michel Zanca, Patrick Canadas, Eric Nativel, Maida Cardoso, Eric Alibert, Guillaume Dupont, Dominique Ambard, Christophe Maurel, Jean-Luc Verdeil, Nadia Bertin, Christophe Goze-Bac, Christophe Coillot

**Affiliations:** 1 L2C, University of Montpellier, CNRS, Montpellier, France; 2 LMGC, University of Montpellier, CNRS, Montpellier, France; 3 CHU Gui de Chauliac, University of Montpellier, Montpellier, France; 4 BPMP, University of Montpellier, CNRS, INRA, Montpellier SupAgro, Montpellier, France; 5 IES, University of Montpellier, CNRS, Montpellier, France; 6 AGAP, University of Montpellier, CIRAD, INRA, Montpellier SupAgro, Montpellier, France; 7 PSH, INRA, Avignon, France; University of Vigo, SPAIN

## Abstract

The climate warming implies an increase of stress of plants (drought and torrential rainfall). The understanding of plant behavior, in this context, takes a major importance and sap flow measurement in plants remains a key issue for plant understanding. Magnetic Resonance Imaging (MRI) which is well known to be a powerful tool to access water quantity can be used to measure moving water. We describe a novel flow-MRI method which takes advantage of inflow slice sensitivity. The method involves the slice selectivity in the context of multi slice spin echo sequence. Two sequences such as a given slice is consecutively inflow and outflow sensitive are performed, offering the possiblility to perform slow flow sensitive imaging in a quite straigthforward way. The method potential is demonstrated by imaging both a slow flow measurement on a test bench (as low as 10 *μm*.*s*^−1^) and the Poiseuille’s profile of xylemian sap flow velocity in the xylematic tissues of a tomato plant stem.

## Introduction

The climate warming implies an increase of stress of plants (drought and torrential rainfall). The understanding of plant behavior, in this context, takes a major importance and flow measurement in plants and trees remains a major issue [1]. This problem could naturally be adressed to Magnetic Resonance Imaging which is well known to be a powerful tool to access water quantity but also flow of liquids. Three types of flow-MRI are commonly used depending on the application [2]: the time of flight, the inflow-outflow and the phase-encoding methods. Flow-MRI techniques are widely used in clinic for flow blood imaging. This field of application, known as MR angiography, concerns rapidly moving flow (typ. ∼10*cm*/*s*) in vessels [3]. In plant biology, the sap flow measurement, is performed by means of the pulse field gradient (PFG) method [4] which belongs to the phase-encoding technique. The PFG method is able to measure the slow velocities of the sap flow (0 − ∼5*mm*/*s* for xylemian flow velocities and ∼0.25 − 0.4*mm*/*s* for phloemian flow velocities, see [5, 6]).

The so-called flip-flop method we present belongs to the inflow-outflow method family. It takes advantage of the flow slice sensitivity using a multi slice spin echo. It is based on the substraction of two multi slices spin echo sequences such as a given slice is both inflow and outflow sensitive giving the possiblility to perform slow flow imaging in a quite straigthforward way.

## Overview of the NMR-MRI flow measurement for plant studies

Nuclear Magnetic Resonance (NMR) is a valuable and non-invasive technique for measuring the liquid flows. The impact of flow velocity in NMR spin-echo measurement has been studied early in the context of slow flows [7]. The decrease in signal from spins moving in both homogeneous and heterogeneous RF coil was used to determine an average spin velocity. The effect of the decrease of the apparent transverse relaxation time ($T_{2}^{0}$) measured in a CPMG (Carr-Purcell-Meiboorn-Gill) sequence was studied while the repetition time of the sequence was much higher than the longitudinal relaxation time (*i.e.* > *T*_1_). The relaxation time difference in the presence of moving water at two different velocities (one of them being null) was then used to retrieve the average velocity (*v*): $\frac{1}{T_{2}^{0}}=\frac{1}{T_{2}}+fvL$, where *T*_2_ is the transverse relaxation time at null velocity, *f* an experimental factor and *L* the RF coil length. Further it was shown on a laboratory experiment simulating a plant stem, that the method can provide access to water flow velocities ranging from 1*mm*/*s* up to 30*mm*/*s* into a 0.8*mm* inner diameter tube. However, the difficulty for obtaining the reference curve at null velocity in this difference method was pointed, even though a some method to solve this issue was proposed by the authors.

Later, a new method was proposed as a way to solve the problem encoutered by Hemminga in their pioneering work. In this method, the use of gradient magnetic field applied in the flow direction makes the spin phase sensitive to the flow velocity [8, 9]. Next, the flow NMR method was improved taking advantage of the NMR imaging combined to dedicated gradient pulse sequence to perform flow imaging into a circular tube [10]. Dedicated pulse field gradient was then designed for microscopic flow imaging [4] and became a standard flow MRI method in plant studies [11].

## Inflow methods

**Fig 1:**
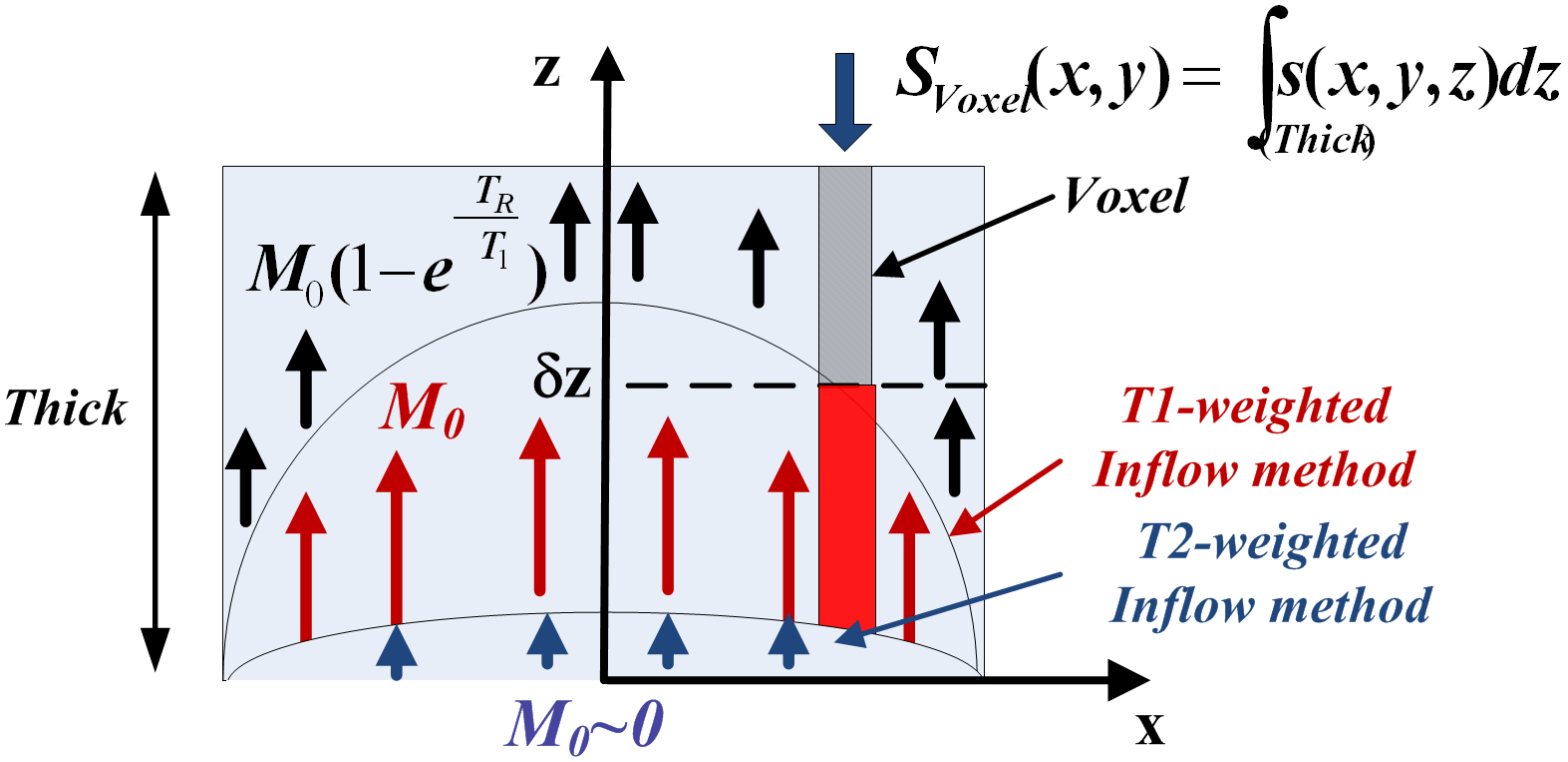
Illustration of the inflow effect. Spins staying within the slice between applications of RF pulses (in black) will not have attained Boltzmann equilibrium (whose magnetization is $M_{0}\left(1-e^{-\frac{T_{R}}{T_{1}}}\right)$); spins entering into the slice (in red) which have not been tilted are saturated (whose magnetization is *M*_0_); spins entering between the *π*/2 pulse and acquisition (in blue) will not be involved in the detected signal (whose magnetization is *M*_0_ ∼ 0).

The inflow effect arises in a selected slice of thickness *T*_*hk*_ in presence of spins moving in a static medium (like tissue). Two situations can be distinguished:

- First, the situation where the spins of the static and flowing liquid have attained Boltzmann equilibrium before entering the slice (*i.e.* the liquid has been in the external magnetic field (*B*_0_, in z direction) for a time much longer than the spin-lattice relaxation time *T*_1_). Following the excitation with a first *π*/2-*π* pulse of a CPMG sequence all the spins will be tilted and detected (*m*_*xy*_ component in the *x* − *y* plane). For the next *π* pulses, the moving spins entering the slice will not produce any signal since they have not been preselected by the *π*/2 pulse. The signal difference with respect to a situation where all the spins remain within the slices can be interpreted as a faster FID decrease, or similarly as a shorter transverse relaxation time. This situation corresponds to the experimental conditions of the Hemminga’s work. It can be referred to as T2-weighted flow MRI.
- Second, the statics spins within the slice are tilted at a repetition time (*T*_*R*_) shorter than the Boltzmann equilibrium time (*T*_*R*_ < 5*T*_1_) while the entering spins are assumed to have attained Boltzmann equilibrium (see Fig 1). The detected signal is thereby increased by the moving spins with respect to a situation where all the spins are statics. The difference in signal between the two situations will be interpreted as a flow signature. It can be referred to as a T1-weighted flow MRI. The method that we present in this article typically concerns this second situation.

We first assume that the velocity within a voxel is unidirectionnal. Inside the voxel, the signal intensity (*S*) results in a certain ratio of moving spins (*α*) entering into the slice and having full magnetization: $M_{0}^{m}$ (assuming that velocity, defined as $\|\vec{v}\|=v$, is sufficiently slow to consider that the spins travelling time in the homogenous magnetic field is sufficient to attain Boltzmann equilibrium (see [2]) and a certain ratio of spins staying in the slice (*β*) which can be decomposed in purely static spins having partial magnetization $M_{0}^{s}\left(1-e^{-\frac{T_{R}}{T_{1}^{s}}}\right)$ and staying moving spins (those which have not escaped the slice between two pulses): $M_{0}^{m}\left(1-e^{-\frac{T_{R}}{T_{1}^{m}}}\right)$. $T_{1}^{m}$ and $T_{1}^{s}$ are longitudinal relaxation times of respectively moving and static spins.

The signal from a certain voxel (*S*) is proportional to the averaged in-plane component of the magnetization (*m*_*xy*_) over the voxel volume ($V_{voxel}=T_{hk}\frac{FOV_{READ}FOV_{PHASE}}{N_{READ}N_{PHASE}}$):

$$\begin{array}{c} S\propto {\;\int \int \int \;}_{V_{voxel}}m_{xy}\left(x,y,z\right)dxdydz, \end{array}$$ (1)

which can be decomposed in two terms:

$$\begin{array}{c} S=k_{c}V_{voxel}\left(\alpha M_{0}^{m}e^{-\frac{T_{E}}{T_{2}^{m}}}+\beta M_{0}^{m}\left(1-e^{-\frac{T_{R}}{T_{1}^{m}}}\right)e^{-\frac{T_{E}}{T_{2}^{m}}}+\left(M_{0}^{s}e^{-\frac{T_{E}}{T_{2}^{s}}}\left(1-e^{-\frac{T_{R}}{T_{1}^{s}}}\right)\right)\right), \end{array}$$ (2)

where $T_{2}^{m}$ and $T_{2}^{s}$ are transverse relaxation times of moving and static, *T*_*R*_ and *T*_*E*_ are repetition and echo times respectively and *k*_*c*_ is the coil sensitivity coefficient [12].

The ratio of moving spins entering the slice just after the *π*/2 RF pulse can be decomposed in two terms: the moving spins entering between the *π*/2 pulse and acquisition time (a fraction $\delta z_{T_{2}}/T_{hk}$ during a time *T*_*E*_), referred to as T2-weighted flow effect, and the moving spins entering between the acquisition time and the following RF pulse (a fraction $\delta z_{T_{1}}/T_{hk}$ during a time *T*_*R*_ − *T*_*E*_), referred to as T1-weighted flow effect.

Consequently, the ratio of moving spins into the slice between two RF pulses (the self-diffusion being neglected [2]) is: $\beta =\left(T_{hk}-\delta z_{T_{1}}-\delta z_{T_{2}}\right)/T_{hk}$. The penetrating distance of T1-weighted moving spins is linked to the velocity: $\delta z_{T_{1}}=v\left(T_{R}-T_{E}\right)$ while the penetrating distance of T2-weighted moving spins, having almost null magnetization, is: $\delta z_{T_{2}}=vT_{E}$. Thus, the fraction of moving spins entering into the slice having full magnetization is: $\alpha =\delta z_{T_{1}}/T_{hk}$.

Then, Eq (2) becomes:

$$\begin{array}{cc} S= & A_{fov}k_{c}(M_{0}^{m}e^{-\frac{T_{E}}{T_{2}^{m}}}(v\left(T_{R}-T_{E}\right)+\left(T_{hk}-vT_{R}\right)\\ & \left(1-e^{-\frac{T_{R}}{T_{1}^{m}}}\right))+M_{0}^{s}e^{-\frac{T_{E}}{T_{2}^{s}}}T_{hk}\left(1-e^{-\frac{T_{R}}{T_{1}^{s}}}\right)), \end{array}$$ (3)

where $A_{fov}=\frac{FOV_{READ}\times FOV_{PHASe}}{N_{READ}\times N_{PHASE}}$ is the voxel surface.

## Flip-flop spin echo multi slice sequence for flow MRI

### Case 1: *T*_*R*_ is not distributed

**Fig 2:**
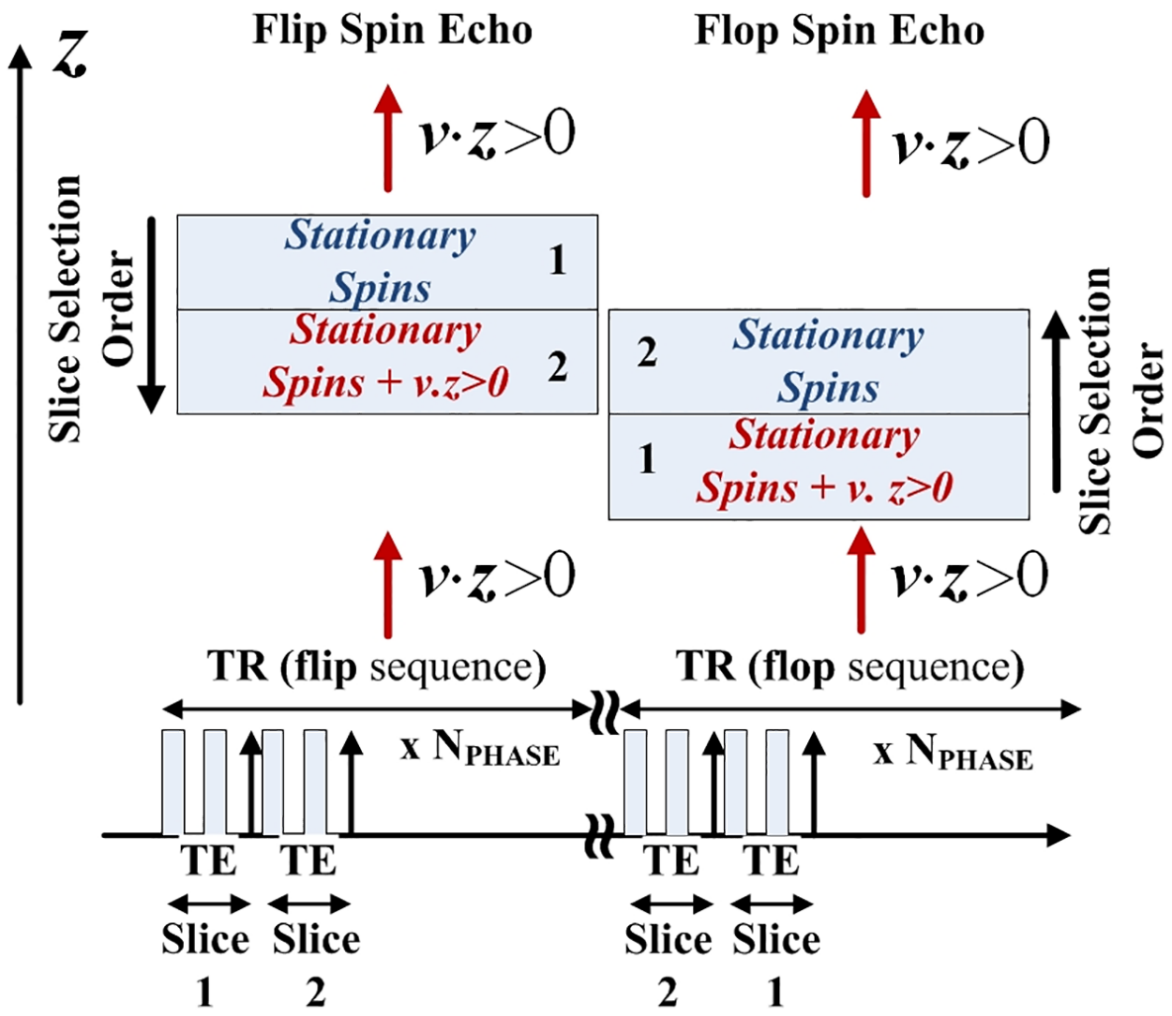
Spin Echo Multi Slice (SEMS) sequence. The upper part of the figure illustrates the inflow measurement depending on the slice location and the slices selection order. The lower part of the figure shows the RF pulse sequence of the SEMS sequence available on the commercial Varian MRI.

The present method takes advantage of the Spin Echo Multi Slice (SEMS) sequence available on Agilent MRI. It will be assumed that the slice acquisitions are performed consecutively (*T*_*R*_ is not distributed) and the time separating two slice acquisitions remains negligible as compared to *T*_*R*_. Depending on the direction of the slice selection (see Fig 2) an enhancement of the signal from slice 2, due to the inflow of saturated spins, will occur in the **flip** sequence (see Fig 2) but not in the **flop** sequence (see Fig 2). The flow is assumed to be mono-directional at the voxel size. If not, the signal between the two flow directions will be partially compensated.

Thus, during the **flip** acquisition, the signal of the second slice will be expressed (assuming a sufficiently slow flow from 2 to 1) as:

$$\begin{array}{cc} S_{flip}= & A_{fov}k_{c}(M_{0}^{m}e^{-\frac{T_{E}}{T_{2}^{m}}}(T_{hk}\left(1-e^{-\frac{T_{R}}{T_{1}^{m}}}\right)\\ & +v\left(T_{R}e^{-\frac{T_{R}}{T_{1}^{m}}}-T_{E}\right))+M_{0}^{s}e^{-\frac{T_{E}}{T_{2}^{s}}}T_{hk}\left(1-e^{-\frac{T_{R}}{T_{1}^{s}}}\right)). \end{array}$$ (4)

Next, during the **flop** sequence the second slice is selected *T*_*E*_ after the first slice. Thus, the moving spins of the second slice (those which stay and those which enter) have almost the same magnetization and the term *α* of Eq (2) vanishes. Nevertheless, there is still a fraction of flow exhibiting negligible magnetization which enters into the slide, due to the T2-weighted flow effect, during *N*_*S*_*T*_*E*_, where *N*_*S*_ is the slice number (in our case *N*_*S*_ = 2). The signal of the second slice will be expressed as:

$$\begin{array}{cc} S_{flop}= & A_{fov}k_{c}(M_{0}^{m}e^{-\frac{T_{E}}{T_{2}^{m}}}\left(T_{hk}-vN_{S}T_{E}\right)\left(1-e^{-\frac{T_{R}}{T_{1}^{m}}}\right)\\ & +M_{0}^{s}e^{-\frac{T_{E}}{T_{2}^{s}}}T_{hk}\left(1-e^{-\frac{T_{R}}{T_{1}^{s}}}\right)), \end{array}$$ (5)

**Fig 3:**
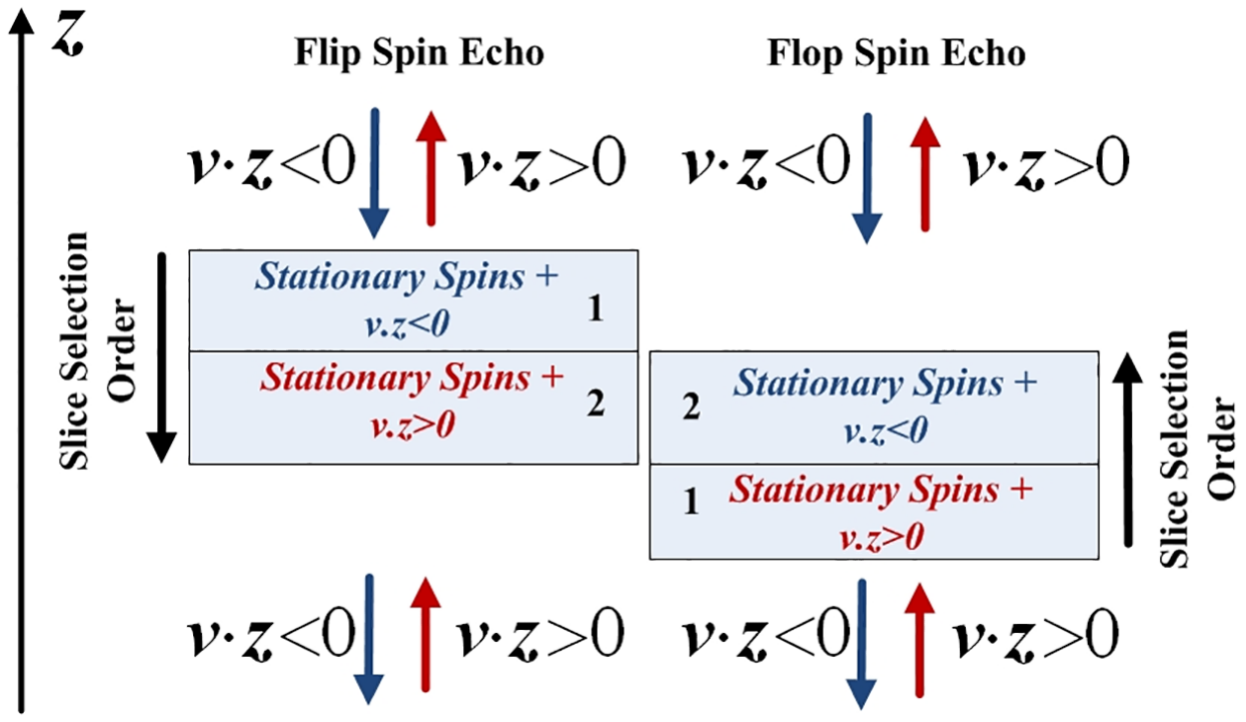
Flip-flop SEMS sequence. In presence of a bidirectionnal flow ($\vec{v}=\pm v\vec{z}$) the second slice of **flip** will be sensitive to the positive direction inflow ($\vec{v}=+v\vec{z}$) while the second slice of **flop** will be sensitive to outflow ($\vec{v}=-v\vec{z}$).

A difference method between the two acquisitions thereby permits to cancel the static spin contribution (from Eqs (4) and (5) while contribution of the moving spins only remains. Lastly, the resulting signal difference, can either be positive or negative depending on the flow direction (as represented in (Fig (3)):

$$\begin{array}{c} S_{flip-flop}=\pm A_{fov}k_{c}M_{0}^{m}e^{-\frac{T_{E}}{T_{2}^{m}}}v\left(\left(T_{R}-N_{S}T_{E}\right)e^{-\frac{T_{R}}{T_{1}^{m}}}+\left(N_{S}-1\right)T_{E}\right), \end{array}$$ (6)

In the following, we will consider experimental conditions as: *N*_*S*_*T*_*E*_ ≪ *T*_*R*_. Thus Eq (6) becomes:

$$\begin{array}{c} S_{flip-flop}≃\pm A_{fov}k_{c}M_{0}^{m}e^{-\frac{T_{E}}{T_{2}^{m}}}vT_{R}e^{-\frac{T_{R}}{T_{1}^{m}}}. \end{array}$$ (7)

In the case of a rapid flow (i.e. such as *v* > *T*_*hk*_/*T*_*R*_) during the **flip** sequence, a part of the flow can cross the second slice and reach the first slice in a time shorter than *T*_*R*_. Thus this part will not contribute to the signal measured in the second slice. In such condition, the flow will be underestimated. To avoid such effect, an estimate of the higher velocity would be needed to design the MRI sequence (in terms of slice thickness or repetition time).

### Case 2: *T*_*R*_ is distributed

We will now consider the case of distributed *T*_*R*_. In this case, slice acquisitions are equally separated at *T*_*R*_/*N*_*S*_. We will assume that *T*_*E*_ ≪ *T*_*R*_/*N*_*S*_. This sequence configuration allows a more efficient spoiler and reduces aliasing from a slice to its neighbour.

During the **flip** acquisition, the signal of the $N_{S}^{th}$ slice will have a similar expression as when *T*_*R*_ is not distributed:

$$\begin{array}{cc} S_{flip}^{bis}= & A_{fov}k_{c}(M_{0}^{m}e^{-\frac{T_{E}}{T_{2}^{m}}}\left(T_{hk}\left(1-e^{-\frac{T_{R}}{T_{1}^{m}}}\right)+vT_{R}e^{-\frac{T_{R}}{T_{1}^{m}}}\right)\\ & +M_{0}^{s}e^{-\frac{T_{E}}{T_{2}^{s}}}T_{hk}\left(1-e^{-\frac{T_{R}}{T_{1}^{s}}}\right)). \end{array}$$ (8)

During the **flop** sequence, the *N*^*th*^ slice is selected at *T*_*R*_/*N*_*S*_ after the (*N*^*th*^ − 1) slice. Thus, a fraction (*vT*_*R*_/*N*_*S*_)/*T*_*hk*_ of moving spins having $1-e^{-\frac{T_{R}/N_{S}}{T_{1}^{m}}}$ fraction of the full magnetization will enter the $N_{S}^{th}$ slice while the remaining fraction of the moving spins (1 − (*vT*_*R*_/*N*_*S*_)/*T*_*hk*_) having $1-e^{-\frac{T_{R}}{T_{1}^{m}}}$ magnetization will stay within the slice. The signal of the $N_{S}^{th}$ slice will be expressed:

$$\begin{array}{cc} S_{flop}^{bis}= & A_{fov}k_{c}(M_{0}^{m}e^{-\frac{T_{E}}{T_{2}^{m}}}(v\frac{T_{R}}{N_{S}}\left(1-e^{-\frac{T_{R}/N_{S}}{T_{1}^{m}}}\right)+\left(T_{hk}-v\frac{T_{R}}{N_{S}}\right)\left(1-e^{-\frac{T_{R}}{T_{1}^{m}}}\right)\\ & +M_{0}^{s}e^{-\frac{T_{E}}{T_{2}^{s}}}T_{hk}\left(1-e^{-\frac{T_{R}}{T_{1}^{s}}}\right)), \end{array}$$ (9)

which becomes:

$$\begin{array}{cc} S_{flop}^{bis}= & A_{fov}k_{c}(M_{0}^{m}e^{-\frac{T_{E}}{T_{2}^{m}}}(T_{hk}\left(1-e^{-\frac{T_{R}}{T_{1}^{m}}}\right)+v\frac{T_{R}}{N_{S}}\left(e^{-\frac{T_{R}}{T_{1}^{m}}}-e^{-\frac{T_{R}/N_{S}}{T_{1}^{m}}}\right)\\ & +M_{0}^{s}e^{-\frac{T_{E}}{T_{2}^{s}}}T_{hk}\left(1-e^{-\frac{T_{R}}{T_{1}^{s}}}\right)), \end{array}$$ (10)

A difference method between the two acquisitions will permit to cancel the static spin contribution (from Eqs (8) and (10)) while only the moving spins contribution will remain. The signal difference will be expressed as:

$$\begin{array}{c} S_{flip-flop}^{bis}≃\pm A_{fov}k_{c}M_{0}^{m}e^{-\frac{T_{E}}{T_{2}^{m}}}vT_{R}\left(\left(1-\frac{1}{N_{S}}\right)e^{-\frac{T_{R}}{T_{1}^{m}}}+\frac{1}{N_{S}}e^{-\frac{T_{R}/N_{S}}{T_{1}^{m}}}\right). \end{array}$$ (11)

### Quantification of the flow velocity

Flip-flop method allows to monitor flow change. This is highly relevant for plants under varying environmental conditions. However, absolute flow measurements would require flow velocity to be calibrated in at least one reference condition. To address this issue we suggest to take advantage of the saturation condition (namely *v* > *T*_*hk*_/*T*_*R*_).

In the case of the flow-MRI in plants we can make some asumptions. Firstly, the xylematic tissues are almost circular and a Poiseuille’s profile is assumed. Secondly, their area is comparable to voxel or, in the best case, to that of several voxels.

In the case of a Poiseuille’s profile, the radial velocity (*v*(*r*)) for a circular xylematic tissue of radius *R* is expressed as: *v*(*r*) = *v*_*max*_(1 − (*r*/*R*)^2^) and the average velocity over the circular area is obtained by calculating:

$$\begin{array}{c} v_{moy}=\frac{1}{\pi R^{2}}\int_{0}^{R}v\left(r\right)2\pi rdr. \end{array}$$ (12)

which leads after some basic computations to: *v*_*moy*_ = *v*_*max*_/2.

In the region where the velocities are such as: *v*(*r*) > *T*_*hk*_/*T*_*R*_, let’s say *r* ∈ [0, *r*_*sat*_]), the velocity signal acquired by the flip-flop method will appear to be saturated at *v*(*r*) = *T*_*hk*_/*T*_*R*_, while the Poiseuille’s profile will appear to be truncated. The saturation condition is reached for *r*_*sat*_ such as:

$$\begin{array}{c} T_{hk}/T_{R}=v_{max}\left(1-{\left(r_{sat}/R\right)}^{2}\right). \end{array}$$ (13)

In this context, Eq (12) can be written:

$$\begin{array}{c} v_{moy}=\frac{2}{R^{2}}\int_{0}^{r_{sat}}\frac{T_{hk}}{T_{R}}rdr+\frac{2v_{max}}{R^{2}}\int_{r_{sat}}^{R}\left(1-{\left(r/R\right)}^{2}\right)rdr. \end{array}$$ (14)

which leads, after some calculations, to:

$$\begin{array}{c} v_{moy}=\frac{T_{hk}}{T_{R}}\left(1-\frac{1}{2v_{max}}\frac{T_{hk}}{T_{R}}\right). \end{array}$$ (15)

The average velocity, whatever the regime, (truncated or not truncated Poiseuille’s profile) will be expressed as a distribution:

$$\begin{array}{c} v_{moy}=H(\frac{T_{hk}}{v_{max}}-T_{R})(\frac{v_{max}}{2})+H(T_{R}-\frac{T_{hk}}{v_{max}})(\frac{T_{hk}}{T_{R}}(1-\frac{1}{2v_{max}}\frac{T_{hk}}{T_{R}})). \end{array}$$ (16)

where *H*(*u*) is the Heaviside step-function such as *H*(*u*) = 0 for *u* < 0 and *H*(*u*) = 1 for *u* > 1.

The latter expression of the average velocity should be either replaced in Eq (7), if *T*_*R*_ is not distributed, or Eq (11), if *T*_*R*_ is distributed. The method will consist in finding the parameter *v*_*max*_ with fits the best to experimental data obtained at different *T*_*R*_ values in a given area (delimited by segmentation). Once *v*_*max*_ is obtained, the running sequence can be performed at *T*_*R*_ such as $T_{R}<\frac{T_{hk}}{v_{max}}$ in order to remain below the truncating regime of Poiseuille’s profile. Then it will be possible to monitor the sap velocity change in response to environmental conditions while the estimate of *v*_*max*_ will allow to put a scale on these changes.

### Optimum parameters of the flip-flop sequence

To determine the optimal parameters of the flip-flop SEMS sequence, we have to maximize the Signal to Noise Ratio (*SNR*, cf. [13]) in a fixed time. The *SNR* is the ratio between the simplified expression of the signal given by Eq (7) and the noise (expressed as $\sqrt{F4kTR_{tot}BW}$, where *F* is the amplifier noise figure, *k* is the Boltzman constant, *T* is the temperature, *BW* is the noise bandwidth and *R*_*tot*_ includes all the noise contibutions: mainly coil resistance and magnetic losses in the sample at the Larmor frequency). The *SNR* of the **flip-flop** SEMS sequence can be simply written as:

$$\begin{array}{c} SNR\propto \frac{S_{flip-flop}}{\sqrt{F4kTR_{tot}BW}}\times \sqrt{AVG}, \end{array}$$ (17)

where *AVG* (for averaging) is the number of acquired images which are typically accumulated to enhance the *SNR*. The optimum parameters have to be chosen for a given acquisition time (*T*_*acq*_ = *AVG* × *T*_*R*_ = *Ct*). In such condition, the *SNR* in a fixed acquisition time is expressed as:

$$\begin{array}{c} SNR\propto \frac{S_{flip-flop}}{\sqrt{F4kTR_{tot}BW}}\times \sqrt{\frac{T_{acq}}{T_{R}}}, \end{array}$$ (18)

By substituting the expression of *S*_*flip*−*flop*_ in Eq (18), this one becomes:

$$\begin{array}{c} SNR\propto A_{fov}k_{c}v\frac{M_{0}^{m}\sqrt{T_{acq}T_{R}}}{\sqrt{F4kTR_{tot}BW}}e^{-\frac{T_{R}}{T_{1}^{m}}}e^{-\frac{T_{E}}{T_{2}^{m}}}. \end{array}$$ (19)

The optimum *T*_*R*_ should satisfy: $\frac{\partial SNR}{\partial T_{R}}=0$, which leads to: $T_{R}=T_{1}^{m}/2$

## Materials and methods

### Flow-MRI: Validation setup

The MRI system comprises a 9.4*T* Varian magnet including gradient coils, the internal available diameter to insert the sample inside the probe is ∼100*mm*. For the demonstration setup, a commercial 43*mm* diameter quadrature birdcage coil (from RAPID biomedical company) dedicated to ^1^*H* imaging was used. The MRI sequence were scheduled under the vnmrj software environment.

**Fig 4:**
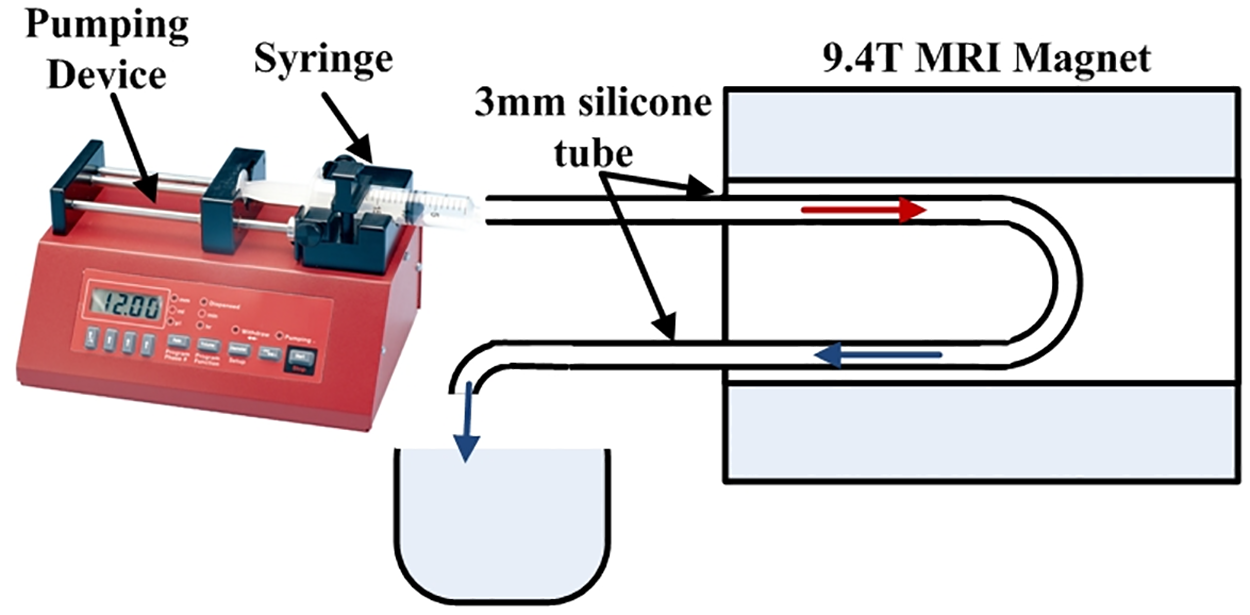
Validation setup description. The Aladdin pumping device pushes the water from the syringe into a silicone tube forming a loop. The silicone tube crossed by opposite directions water flows is inserted within the birdcage coil into the MRI magnet.

The flow experimental setup consists of a nanoliter Aladdin syringe pump device (AL1000 from World Precision Instruments company) pushing a water filled syringe (14mm diameter) generating a water flow through a silicone tube of 3*mm* internal diameter. The silicon tube makes a loop in order to create opposite flow velocities. The assembly is installed inside the birdcage coil into the magnet as represented on Fig 4 (N.B. the birdcage coil is not represented).

### Flow-MRI: Tomato plant setup

An home made RF saddle-coil (diameter = 14*mm*, height = 20*mm* and 1mm diameter copper wire) was constructed. The RF saddle-coil can be open in order to welcome the stem to be imaged. The copper conductor wire constituting the RF saddle-coil is segmented in two portions of equal length (such as wire length < λ/8 to remain under quasi-static hypothesis) separated by a segmentation capacitance *C*_*s*_ = 2*pF*. The RF saddle-coil, used both to excite and to detect the spins, is associated with an home made tuning-matching circuit based on existing designs [12, 14, 15] and electromagnetically shielded by means of copper tape.

### Plant material and cultivation

**Fig 5:**
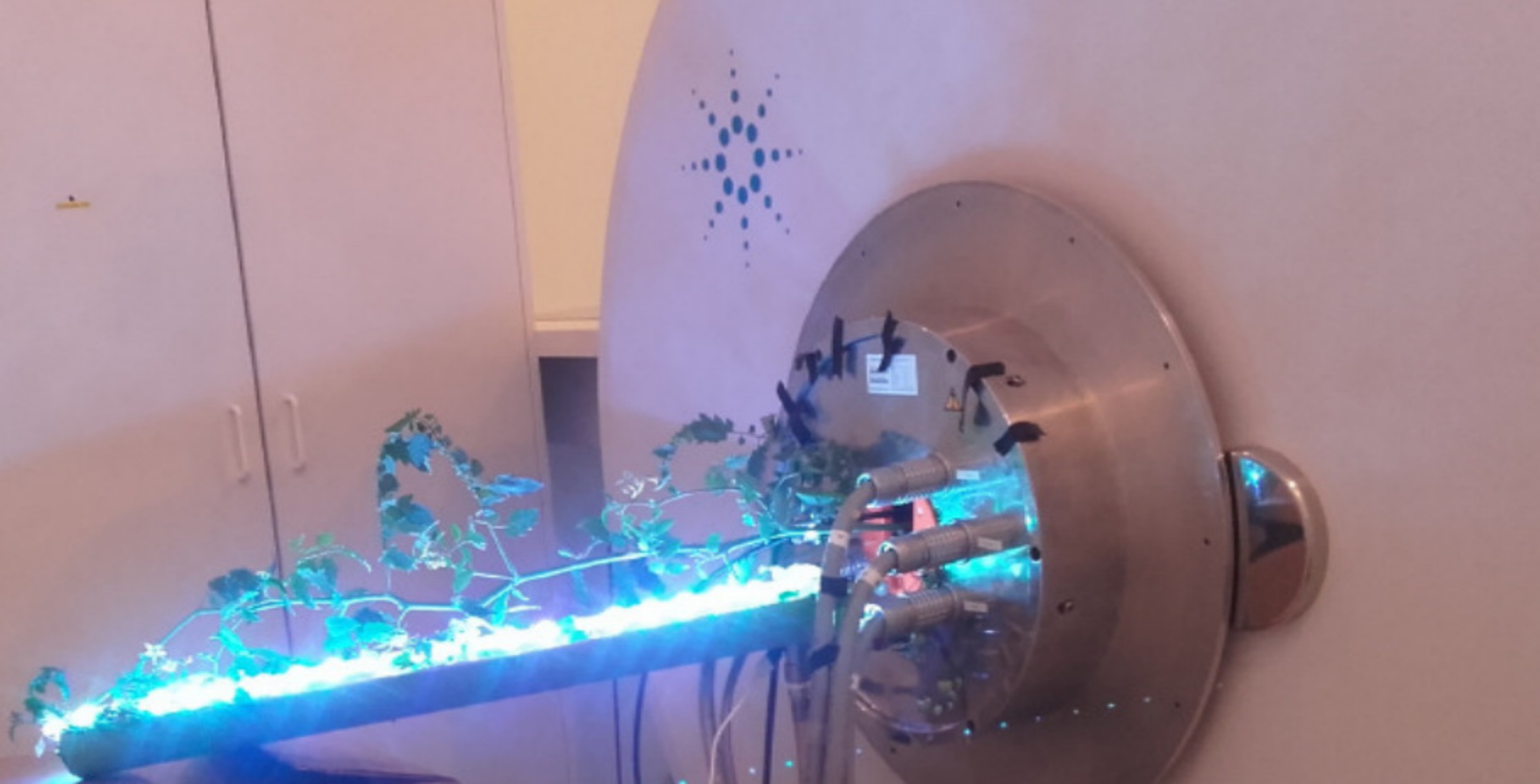
Tomato plant installation. The tomato plant is installed inside the MRI magnet. The external part of the plant is enlightened by the LED panel.

Tomato plants cv. WVA106 were grown on rockwool cubes (10*cm* × 10*cm* × 10*cm*) under greenhouse conditions. All side shoots were removed and flowers were pollinated by-hand three times a week. Plants were supplied daily with a nutrient solution (Liquoplant Rose, Plantin, Courthézon, France) diluted between 0.4 ‰ and 0.8 ‰ according to the plant development stage, which corresponds to an average electroconductivity of 1.8*mS*.*cm*^−1^. The volume and frequency of irrigation were monitored in order to match the evapostranpiration demand. After fruit setting of the fifth truss, one plant was transferred to the lab and adapted to the prevailing ambient conditions for 2 days. Nutrient solution was supplied during this period. Then the plant and the coil were inserted vertically into the MRI magnet. The RF saddle-coil was installed on the lower part of the stem, between the collar stem and the first fruit truss. The environmental conditions in the magnet were 20 ± 1^*o*^*C* at daytime and night-time. A light source using four stripes of LED panel type SMD5050 was installed inside an half circular epoxy tube in order to lighten the canopy of the tomato plant (see Fig 5). The stripes LED has a density of 3 LEDs per 10cm and each LED produces ∼16*lumens*.

## Results

### Qualitative flow imaging

**Fig 6:**
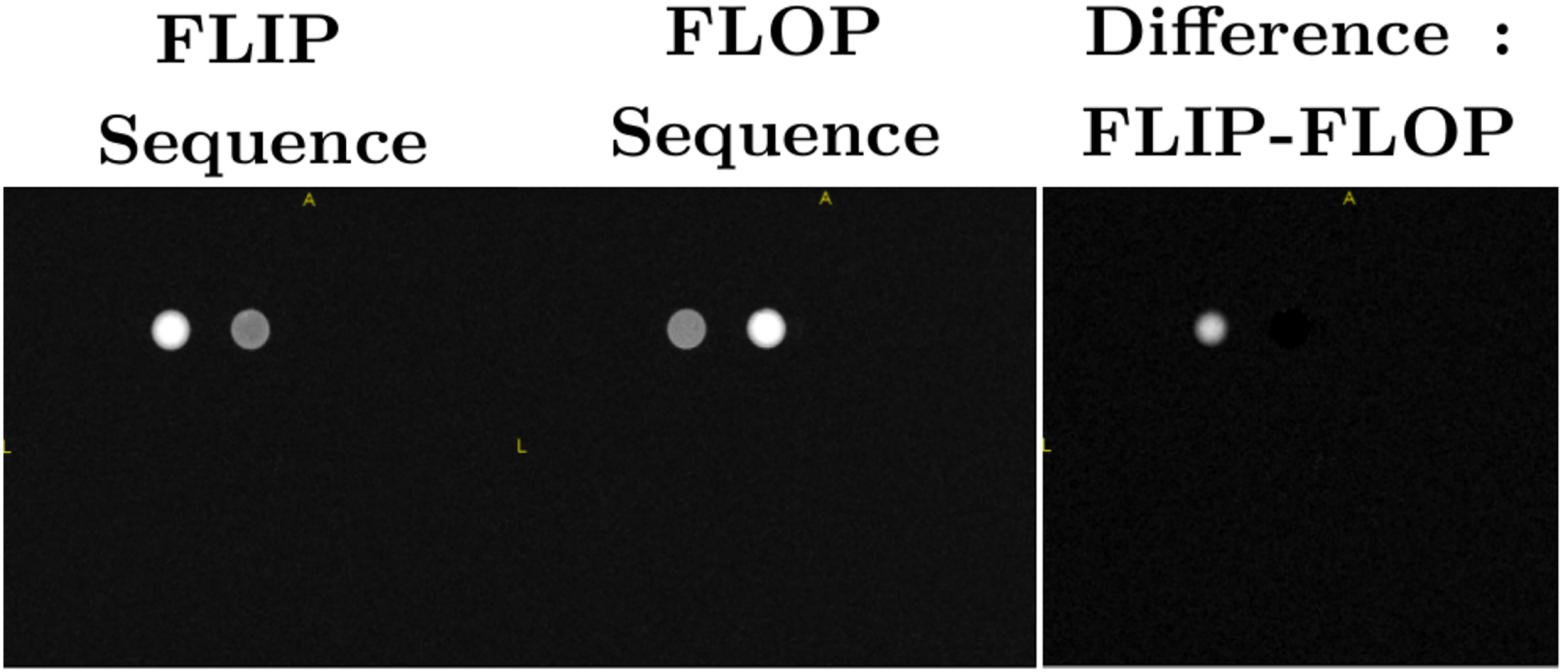
Flip-flop MRI sequence acquisition. From left to right: flip sequence, flop sequence and substracted image (*flip* − *flop*). Experimental conditions: flowing water in a 3mm inner diameter silicone tube, *v*_*mean*_ = 120*μm*/*s*, *T*_*R*_ = *T*_1_/2 = 1.23*s*, *T*_*E*_ = 15*ms*, *FOV*_*READ*_ = 20*mm*, *FOV*_*PHASE*_ = 20*mm*, *N*_*READ*_ = *N*_*PHASE*_ = 128, *T*_*hk*_ = 1*mm*, 4 Slices and *T*_*acq*_ = 2 × 2*min*38.

According to the described flip-flop method, two SEMS sequences with a common slice and opposite directions of the slice selection order have been implemented. The setup is presented in Fig 4. The longitudinal relaxation time of the fluid (pure water in our experiment) was first identified: $T_{1}^{m}=2.46s$. The flowing water imposed by the pumping device in this experiment was *v*_*mean*_ = 100*μm*/*s*. The acquired images (flip, flop and substracted (= *flip* − *flop*)) are shown on Fig 6. The intensity increase due to the flow on both *flip* and *flop* acquisition is difficult to distinguish from the static spin signal (see flip and flop images of Fig 6) while the substraction of the two acquired images (as depicted on the *flip* − *flop* image of Fig 6) clearly shows an hyper signal due to the inflow in the left tube and an hypo signal due to the outflow in the right tube.

**Fig 7:**
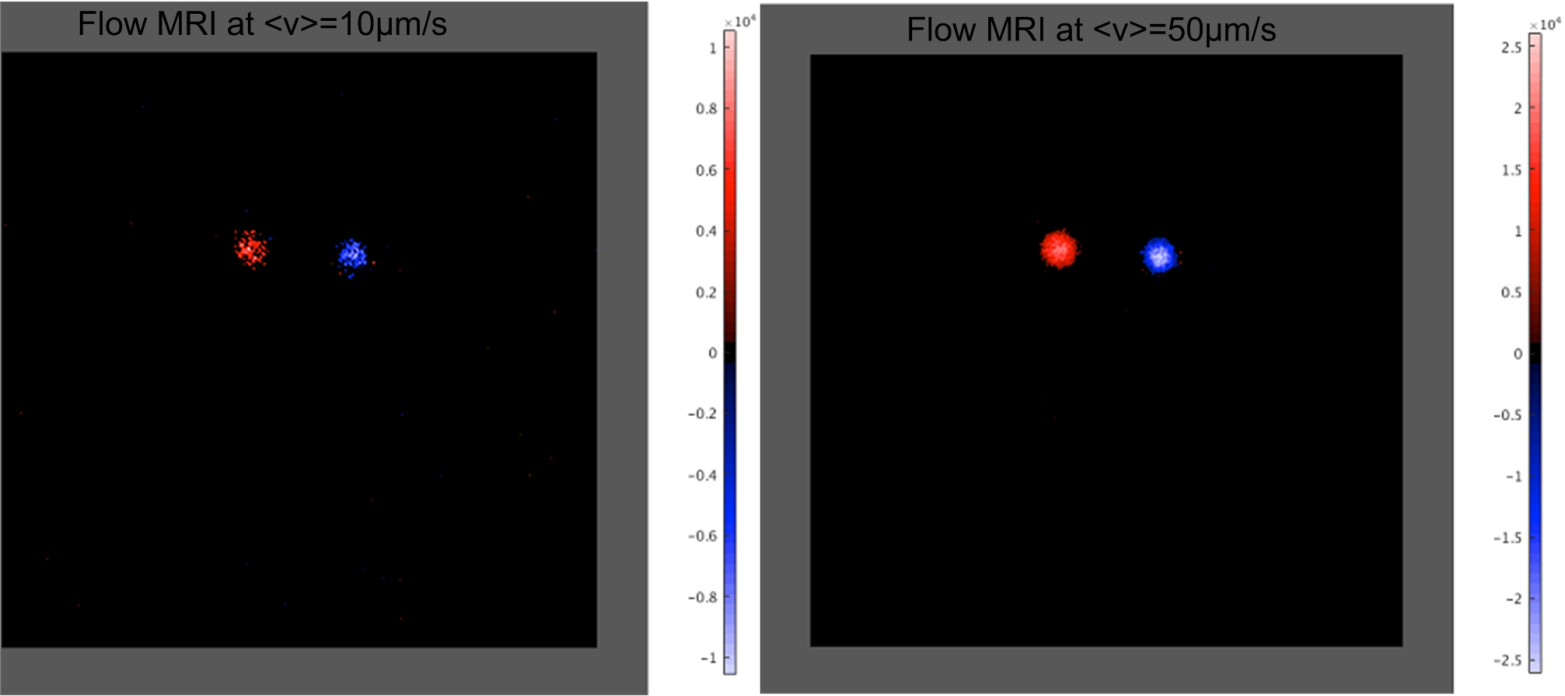
Flip-flop flow-MRI. Right figure: at 10*μm*/*s* and left figure: 50*μm*/*s*. The following parameters were used: *T*_*R*_ = 1.2*s*, *T*_*E*_ = 15*ms*, *FOV*_*READ*_ = 40*mm*, *FOV*_*PHASE*_ = 40*mm*, *N*_*READ*_ = *N*_*PHASE*_ = 256, *T*_*hk*_ = 0.5*mm*, 2 slices, *AVG* = 2 and *T*_*acq*_ = 2 × 10*min*14.

The flip-flop method was next evaluated at very slow velocities: 10*μm*/*s* and 50*μm*/*s* within a 0.16*mm* × 0.16*mm* × 0.5*mm* voxel in ∼20*min*. The flow images so-obtained, shown in (Fig 7), illustrate the ability of the method to perform a good qualitative imaging of extremely slow-flow and to discriminate flow directions within the same slice. The signal intensity (see Fig (7)) is positive or negative in the right and left tube, respectively. These opposite signs are obviously related to the flow direction imposed by the setup configuration. The profile of the signal intensity exhibits a parabolic profile which corresponds to the Poiseuille’s profile expected in a circular tube.

### Quantitative flow imaging

**Fig 8:**
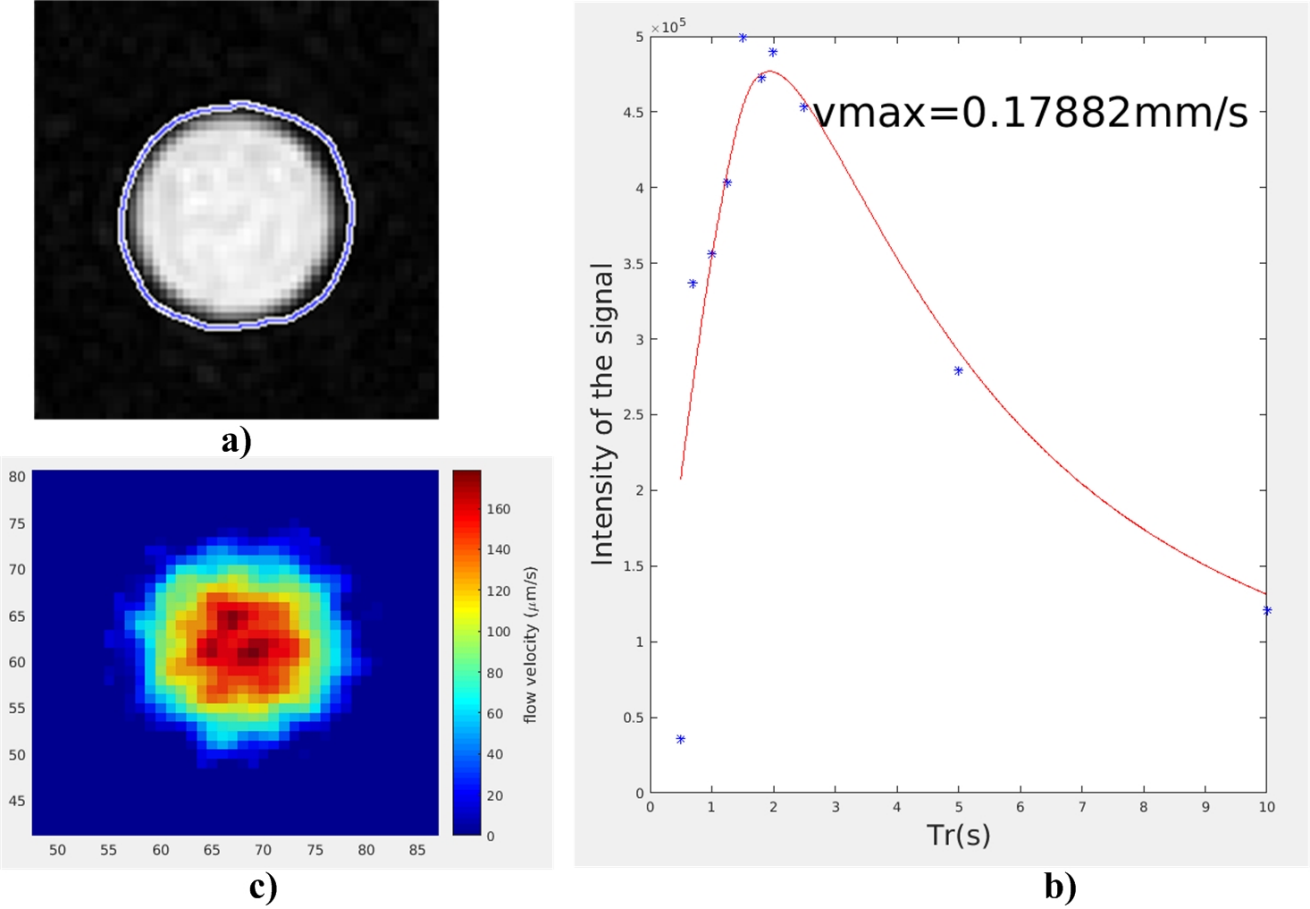
Flow velocity quantification. a) A segmentation is applied on a region crossed by an assumed monodirectional flow. b) The formula given by Eq (16) is applied to the set of *T*_*R*_ experiment to get the best *vmax* fitting value (here, 0.178*mm*/*s* while the expected value would be 0.2*mm*/*s*). c) The velocity scale can be added to the flow image.

The quantification method described in the previous section was implemented to quantify the intensity of the flow imaging obtained by flip-flop method. The result is reported on (Fig 8). It shows that the image scaled using the fitting method remains in good agreement with expected value for an ideal Poiseuille flowing (*v*_*max*_ ∼ 0.2*mm*/*s*) while a Poiseuille’s profile is obtained.

## Xylemian sap flow imaging on tomato plant stem

**Fig 9:**
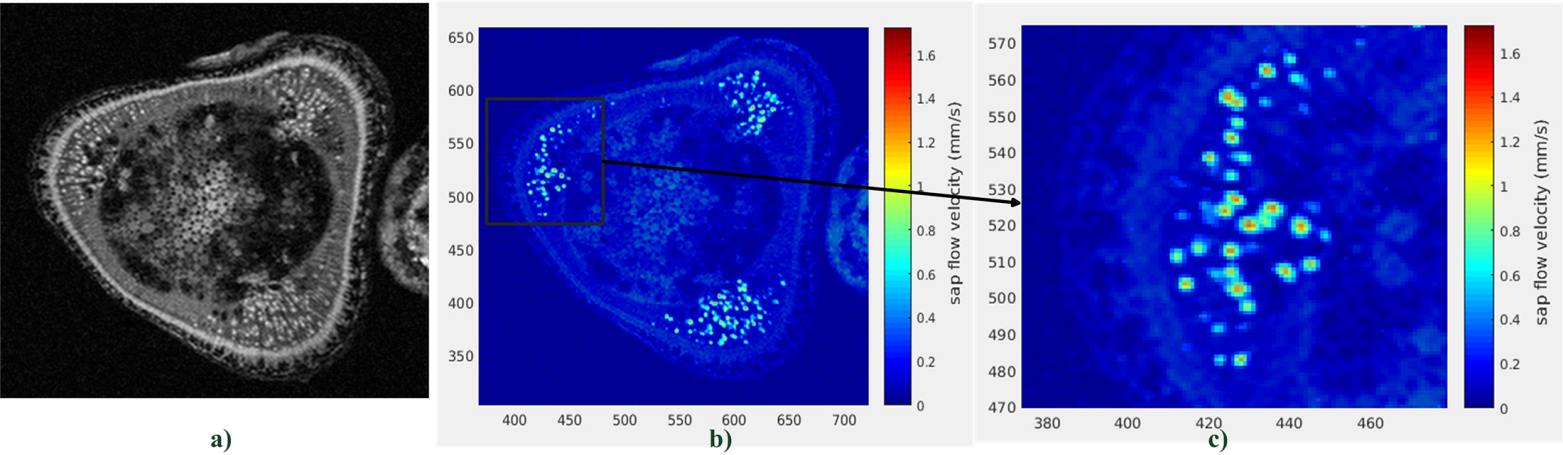
Illustration of xylemian sapflow imaging on tomato stem. a) Anatomical image acquisition (*T*_*R*_ = 1.2*s*, *T*_*E*_ = 15*ms*, *FOV*_*READ*_ = 40*mm*, *FOV*_*PHASE*_ = 40*mm*, *N*_*READ*_ = *N*_*PHASE*_ = 256) b) Superimposition of the anatomical acquisition (background) with the flow acquisition (only the active xylematic tissues appear) c) Zoom on an active xylematic tissue region showing an higher intensity at the center of individual xylematic tissues.

The flip-flop flow-MRI method was used to monitor the xylem sap flow on the stem of the tomato plant using the home built RF saddle-coil. Experiments were initially run at different *T*_*R*_ values (from 100*ms* up to 2*s*). The evolution of the average signal on a xylematic tissue at different *T*_*R*_ was then used to deduce the max velocity by the fitting method described previously (under the assumption of a truncated Poiseuille’s profile). Thus, maximum velocity about 1.6*mm*/*s*, at the center of a single xylematic tissue, was determined allowing to put a scale on the flow-MRI image (see Fig 9b and 9c).

## Discussion

The flip-flop method is easy to handle with commercial MRI system having multi slice spin echo sequence. The flip-flop method provides a qualitative velocity-weighted image without data processing. The absolute quantification is obtained from a trivial data processing. These considerations seems advantageous with respect to the flow-MRI in plants using the PFG method [4]. However, the flip-flop method can suffer of some drawbacks highly dependent on hardware flaws. On one side, asymmetrical gradients can induce errors in voxel location between flip and flop acquisition, which in turns results in an offset after image subtractions. In our MRI system, this artefact was cancelled by selecting centered slices at (0,0). On the other side, in principle the flip-flop signal (see Eq 7) should not be dependent of the thickness of the selected slice (under the slow flow regime assumption, namely *vT*_*R*_ < *T*_*hk*_) while, in practice, we noticed a decrease in signal for thick slices. This effect can be attributed to an imperfect selection of the slice’s profile. Due to this effect, the flip-flop flow-MRI method should be preferably used for thin slices (< 1*mm*).

Finally, the major criterion for the comparison between flip-flop method and the well-known PFG method [4] would be their respective sensitivity. On one side, the flip-flop signal is proportionnal to a fraction (∝ *vT*_*R*_) of the inflow entering into the slice while the PFG methods will take advantage of multiple *N* echos acquisition but a smaller fraction of the inflow (∝ *vNT*_*E*_, *N* being the echo number) since *NT*_*E*_ remains smaller than *T*_*R*_. Thus, the sensitivity comparison remains an open question. However, a promising aspect would be the complementary between these two methods which could lead to a fruitful combination.

In order to perform long-term monitoring for agronomic studies, the flip-flop flow-MRI acquisition should be repeated to follow the relative change of the xylemian’s flow dynamic. In the actual setup configuration, it must be noted that the plant is clearly under sub-optimal condition which is the plausible reason of the slower xylemian velocities than those reported in reference works [5].

## Conclusion

The *T*_1_ weighted flow phenomenon used in the flip-flop method offers a simple way to perform flow MRI whereby two multi slices spin echo sequence are combined and slice selections are done in reverse direction within the same repetion time. The flow measurement is obtained by substracting the same slice in the two acquisitions. It gives an image qualitatively related to the water flow and its direction while the signal from the stationary spins is suppressed. It allows to detect flow as slow as few 10*μm*/*s* in a short acquisition time (few tens of min in the reported case). The method has the interesting property of providing direct qualitative flow imaging without any calculation.

The quantitative scale of flow velocity image is obtained by means of a fitting method taking advantage of the thickness signal saturation under the hypothesis of a Poiseuille’s profile.

The method efficiency was demonstrated through the accurate imaging of a sap flow in the xylematic vascular system in the stem of a tomato plant.

These features make the flip-flop method highly relevant and efficient to monitor sap flow dynamics and variations in plants.
